# First-generation and preclinical evaluation of an EphA5-targeted antibody-drug conjugate in solid tumors

**DOI:** 10.1172/JCI188492

**Published:** 2025-07-15

**Authors:** Fernanda I. Staquicini, Fenny H.F. Tang, Vanessa de Oliveira, Sun-Young Kim, Ethan R. Chen, Christopher Markosian, Daniela I. Staquicini, Yongjian Wu, J. Kellogg Parsons, Kirstin F. Barnhart, Stephen C. Alley, Isan Chen, Wadih Arap, Renata Pasqualini

**Affiliations:** 1 MBrace Therapeutics, San Diego, California, USA.; 2 Rutgers Cancer Institute, Newark, New Jersey, USA.; 3 Division of Cancer Biology, Department of Radiation Oncology, and; 4 Division of Hematology/Oncology, Department of Medicine, Rutgers New Jersey Medical School, Newark, New Jersey, USA.

**Keywords:** Oncology, Therapeutics, Cancer, Cancer immunotherapy

## Abstract

Contemporary cancer treatment strategies are shifting toward targeted therapies to improve efficacy and minimize toxicity. Here, we report the design and preclinical evaluation of MBRC-101, a first-in-class antibody-drug conjugate (ADC) targeting EphA5, a receptor tyrosine kinase with an established role in embryonic development but not extensively studied in cancer. We show that EphA5 is expressed in multiple solid tumors, including cancers of the aerodigestive (non–small cell lung, head and neck, gastric, colon, and pancreatic) and genitourinary (bladder and ovary) tracts, as well as most breast cancer subsets (including triple-negative tumors), with limited expression in normal tissues. MBRC-101 is a humanized anti-EphA5 antibody conjugated to monomethyl auristatin E (MMAE) through a ThioBridge, thereby ensuring stable drug-to-antibody ratio and reducing off-target effects. MBRC-101 showed potent antitumor activity, achieving complete tumor regression in several patient-derived xenograft models. Preclinical Good Laboratory Practice–compliant toxicology studies in rats and nonhuman primates demonstrated that MBRC-101 is well tolerated, with observed toxicities limited to known MMAE off-target effects. These findings establish EphA5 as a therapeutic target in cancer and support the translational development of MBRC-101 as a promising ADC candidate for clinical evaluation, currently in a first-in-human multicenter investigational trial for patients with advanced solid tumors (ClinicalTrials.gov, NCT06014658).

## Abstract 2

We describe a new target for cancer therapy and developed an antibody-based therapeutic currently in a first-in-human clinical trial for patients with advanced solid tumors

## Graphical abstract

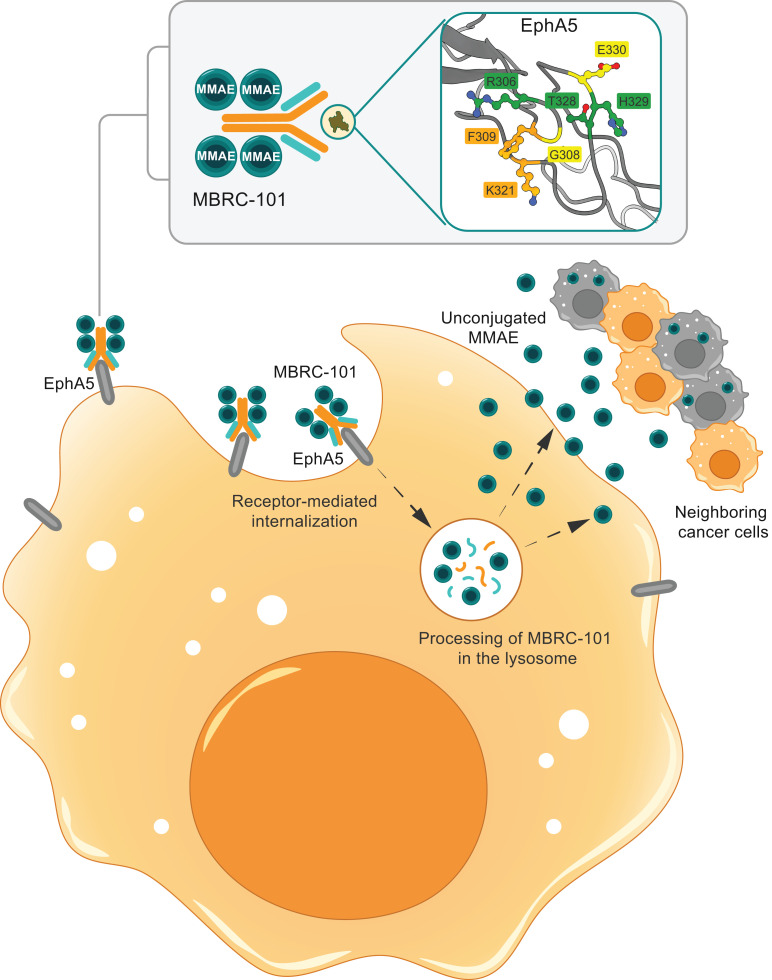

## Introduction

Nonspecific, cytotoxic, drug-based systemic chemotherapy still anchors most treatment regimens for a broad range of cancers. These agents, however, have very low therapeutic indices and often generate severe side effects, thereby restricting their effective use in patients with cancer. In contrast, antibody-drug conjugate (ADC) agents use a targeting mAb component to deliver cytotoxic payloads directly to cancer cells while sparing normal tissues.

Only 5 cancer-specific targets have thus far been approved by the FDA for ADC-based therapy against solid tumors: human epidermal growth factor receptor 2 (1, 2), human trophoblast cell surface glycoprotein antigen 2 (TROP2) (3, 4), tissue factor (5, 6), nectin cell adhesion molecule 4 (nectin-4) (7, 8), and folate receptor α (9, 10). Therefore, the discovery and validation of new molecular targets for ADC applications constitute a promising strategy to advance cancer biology and the emerging field of targeted cancer therapy.

Here we report the receptor tyrosine kinase EphA5 as a highly selective target for directed anticancer therapy (11, 12). We demonstrate that EphA5 is expressed in many human solid tumors, including aerodigestive tract tumors (non–small cell lung [NSCLC], head and neck, gastric, colon, and pancreatic cancers), genitourinary tract tumors (bladder and ovarian cancers), and most subsets of breast tumors (including triple-negative cancer). In contrast, EphA5 displays no or limited expression in normal human tissues.

We designed, generated, and developed MBRC-101, a first-in-class targeted ADC against EphA5, and conducted a comprehensive preclinical program of efficacy and Good Laboratory Practice (GLP) toxicology to support investigational testing in human patients with cancer. MBRC-101 is an ADC composed of a humanized anti-EphA5 antibody conjugated to a monomethyl auristatin E (MMAE). MBRC-101 uses the clinically validated valine-citrulline cleavable linker and a proprietary ThioBridge disulfide rebridging conjugation technology to form a homogeneous ADC with 4 drug molecules per antibody (drug/antibody ratio of 4 [DAR4]). MBRC-101 was highly active when tested in patient-derived xenograft (PDX) models of human cancer expressing moderate to high levels of EphA5, potently regressing tumor xenografts relative to controls in all experimental designs. Toxicologic evaluation in rats and nonhuman primates showed that MBRC-101 is well tolerated with dose-proportional toxicokinetics. Collectively, these results demonstrate that EphA5 is a viable molecular target for the development of mAb-based therapies, and that MBRC-101 has many key attributes of a promising ADC candidate for the treatment of human solid tumors.

## Results

### EphA5 as a target for antibody-based therapies.

**Figure 1:**
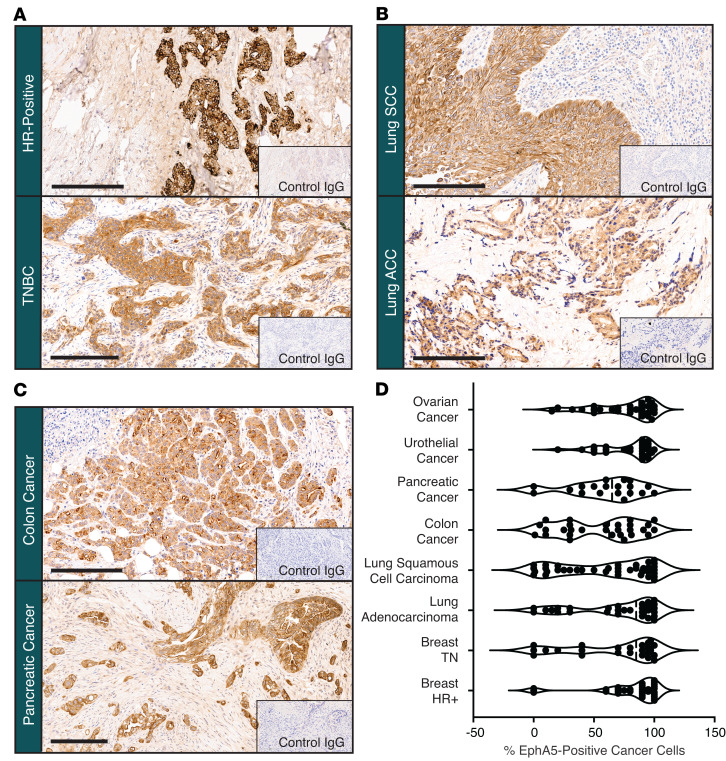
EphA5 expression in multiple cancer indications. Illustrative images of EphA5 expression in (**A**) breast, (**B**) lung, and (**C**) colon and pancreatic cancers. In all cases, EphA5 expression is observed in both the cytoplasm and on the membrane of cells, with expression restricted to cancer cells and not the surrounding adjacent tissue. (**D**) Percentage of EphA5-expressing cancer cells detected in archival tumor samples. Scale bar, 200 μm. Data presented as mean ± SEM. ACC, adenocarcinoma; HR, hormone receptor; SCC, squamous cell carcinoma; TNBC, triple-negative breast cancer.

EphA5 is a receptor tyrosine kinase that belongs to the large Eph receptor family. Eph receptors use cell-cell interactions (along with their corresponding ephrin ligands) to regulate a diverse array of normal physiologic processes, including cell morphology, adhesion, movement, proliferation, survival, differentiation, and axonal guidance, among other functions (13). Expression of EphA5 in cancer has been documented in NSCLC (11), ovarian (14), and pancreatic (15) cancers. We developed and optimized an IHC method to detect the expression of EphA5 in archival tissue sections of various human cancers and corresponding normal tissues. We confirmed the expression of EphA5 in lung, ovarian, and pancreatic cancers and found EphA5 is also expressed in several other human solid tumors, including 84% of breast (estrogen-receptor-positive, *n* = 23 of 26 [88%]; triple-negative breast cancer [TNBC], *n* = 18 of 23 [78%]), 70% of gastric (*n* = 7 of 10), and 68% of colon (*n* = 15 of 22) cancers, among others (Figure 1).

We detected selective expression of EphA5 on the membrane and in the cytoplasm of cancer cells of both estrogen receptor-positive and TNBC (Figure 1A), lung squamous cell carcinomas, and lung adenocarcinomas (Figure 1B), as well as colon, pancreatic, gastric, ovarian, and urothelial cancers (Figure 1, C and D). In most cases, the percentage of EphA5-expressing cancer cells per tissue section averaged 70% or higher, except for colon cancer, in which approximately 45% of cancer cells showed positive EphA5 staining. Intensity of staining was moderate in most sections, with many (but not all) cells demonstrating a clear discernible membranous pattern. H-scores for cytoplasmic and membranous staining were determined for each tumor type and ranged from 0 to 290, with most cases scoring at moderate levels (>100–200). EphA5 expression was not detected in adjacent, nonmalignant tissues in any of the tumors examined. In nonmalignant tissue samples (*n* = 35 normal adult tissues), presence of EphA5 on the membrane and in cytoplasm of normal cells was restricted to the urothelial mucosa of the urinary bladder and ureter, the tubal epithelium of the fallopian tube, and the foveolar mucosa of the stomach. Similar staining patterns were detected in relevant normal tissue sections from nonhuman primates and the percentage of EphA5^+^ cells ranged from 10% to 100% (Supplemental Figure 1, A and B; supplemental material available online with this article; https://doi.org/10.1172/JCI188492DS1).

**Figure 2:**
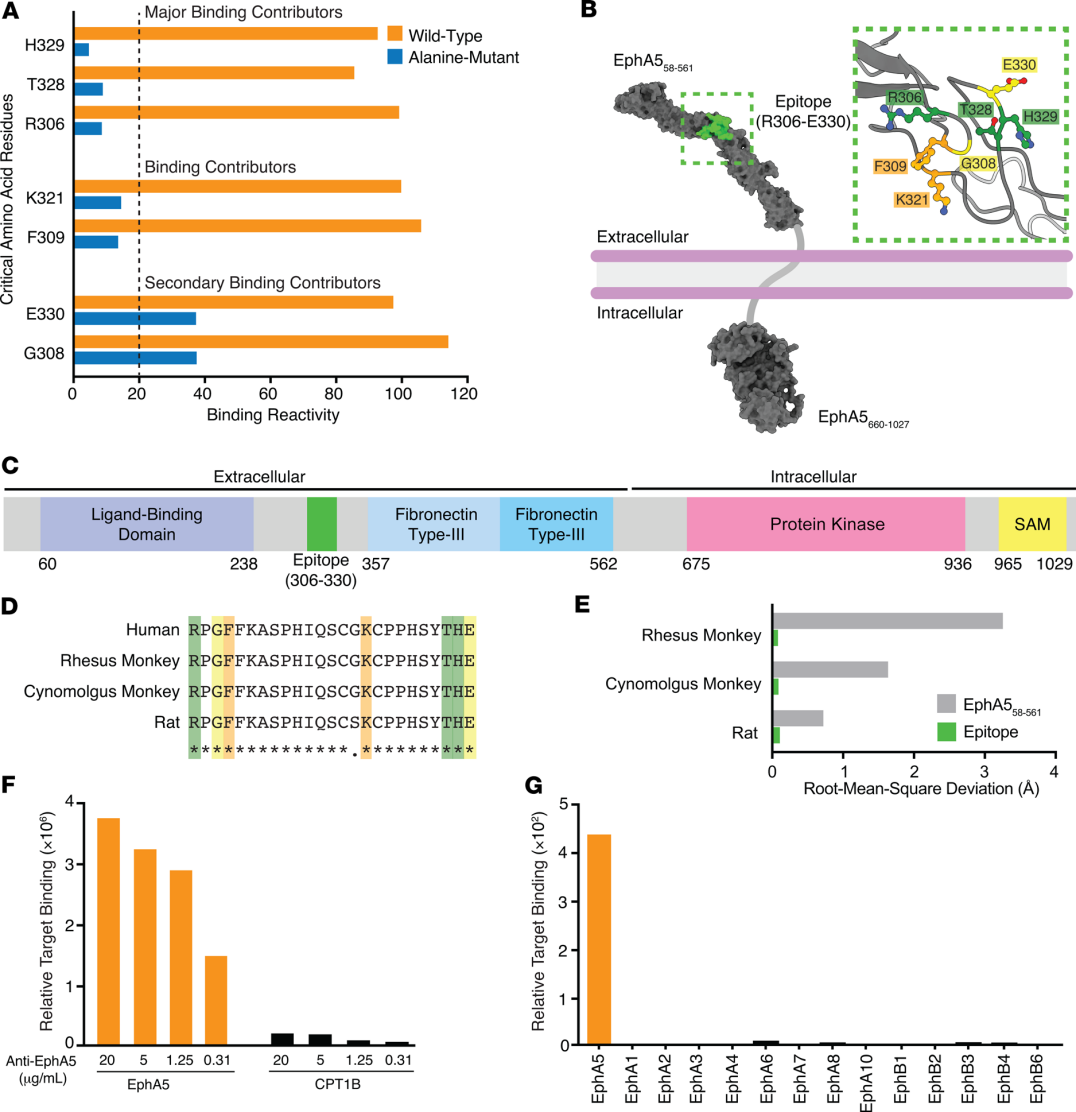
Identification and structural analysis of the binding epitope recognized by the anti-EphA5 antibody. (**A**) Antibody-binding epitope of EphA5 mapped by site-directed alanine scanning. (**B**) AlphaFold-predicted, 3-dimensional, atomic-level structure of full-length human EphA5 containing the identified epitope (green) and excluding low-confidence regions. Consensus protein topology prediction was determined by TOPCONS. The inset visualizes the critical amino acid residues responsible for binding to the anti-EphA5 antibody (green, major binding contributor; orange, binding contributor; yellow, secondary binding contributor). (**C**) Schematic representation of the identified epitope (residues R306–E330) relative to the established domains of human EphA5. (**D**) Multiple sequence alignment of the epitope (residues R306–E330) derived from relevant EphA5 orthologs (green, major binding contributor; orange, binding contributor; yellow, secondary binding contributor). (**E**) Conformational similarity between the extracellular region (residues S58–P561) or epitope (residues R306–E330) of human EphA5 with its corresponding ortholog following best-fit superimposition of AlphaFold-predicted 3-dimensional atomic-level structures. Root mean square deviation was calculated for paired Cα atoms of all corresponding residues. (**F**) MPA confirmed dose-dependent binding specificity of the anti-EphA5 antibody to EphA5. (**G**) All other members of the Eph family of receptors were evaluated and did not show cross-reactivity with the anti-EphA5 antibody. SAM, sterile alpha motif.

The high prevalence of EphA5 in solid tumors and the restricted expression in normal tissues supported the development of a EphA5-targeted, antibody-based therapy to treat human cancers. We first designed and generated a humanized anti-EphA5 antibody and next characterized its binding epitope by using high-throughput scanning site-directed mutagenesis. We constructed an EphA5 mutant library, in which 1 of every individual amino acid residue of the human EphA5 protein sequence was site mutated to alanine, expressed on the surface of HEK293 cells, and individually tested for binding in high-throughput flow cytometry assays. Binding of the anti-EphA5 antibody to each alanine mutant clone was displayed as mean binding reactivity (Figure 2A). Nonmutated (wild-type) EphA5 was used as a control. Decreased binding activity (to < 20% of control) was set as an arbitrary threshold to identify critical residues whose site mutations generated the lowest binding reactivity and were likely to confer the highest energetic contributions to antigen-epitope binding. Residues R306, T328, and H329 were designated as critical for binding (to < 10% of control). Residues K321 and F309 were identified as binding contributors (to < 20% of control), whereas residues G308 and E330 showed moderate reduction in binding but were secondary binding contributors due to their proximity to the major residues.

After the identification of key residues of EphA5 for antibody recognition, we further characterized the binding epitope at the atomic level. Consensus protein topology prediction with TOPCONS (http://topcons.net/) determined that the sole transmembrane region of human EphA5 comprises residues I575–S595. The N- and C-termini can be found in the extracellular and intracellular spaces, respectively, providing a basis for extracellular recognition of the epitope (residues R306–E330) by the anti-EphA5 antibody. We next visualized the identified epitope, including the critical residues, in the 3-dimensional, atomic-level structure of full-length human EphA5, as predicted by AlphaFold (Figure 2B). In the context of established domains of EphA5, the epitope is located within the cysteine-rich linker between the ligand-binding domain and the first fibronectin type-III repeat (Figure 2, B and C) (16). We also determined the evolutionary conservation of the epitope in terms of sequence and structure. The 25-residue epitope is fully (100%) or nearly entirely (96%) conserved in amino acid sequence across EphA5 orthologs of relevant nonclinical species, without any variation in the critical residues (Figure 2D). Moreover, the epitope maintains a high degree of predicted conformational similarity across these species, as indicated by the minimal root mean square deviation (RMSD) of paired C_α_ atoms (Figure 2E). Altogether, these findings indicate that the epitope is found in the extracellular region of EphA5 and is conserved across relevant species, serving as a robust foundation for nonclinical toxicology studies in these animal models.

We next used the membrane proteome array (MPA) (17) to profile the specificity of our anti-EphA5 antibody against more than 6,000 human membrane proteins individually expressed on HEK293 cells. The anti-EphA5 antibody bound to EphA5 and carnitine O-palmitoyltransferase 1 (but to a far lesser extent), which is an intracellular protein exclusively found on the outer membrane of mitochondria (Figure 2F). Notably, all other members of the Eph and ephrin families were expressed in the cell array and did not show any cross-reactivity with EphA5 (Figure 2G), an auspicious sign of specificity confirmation.

**Figure 3:**
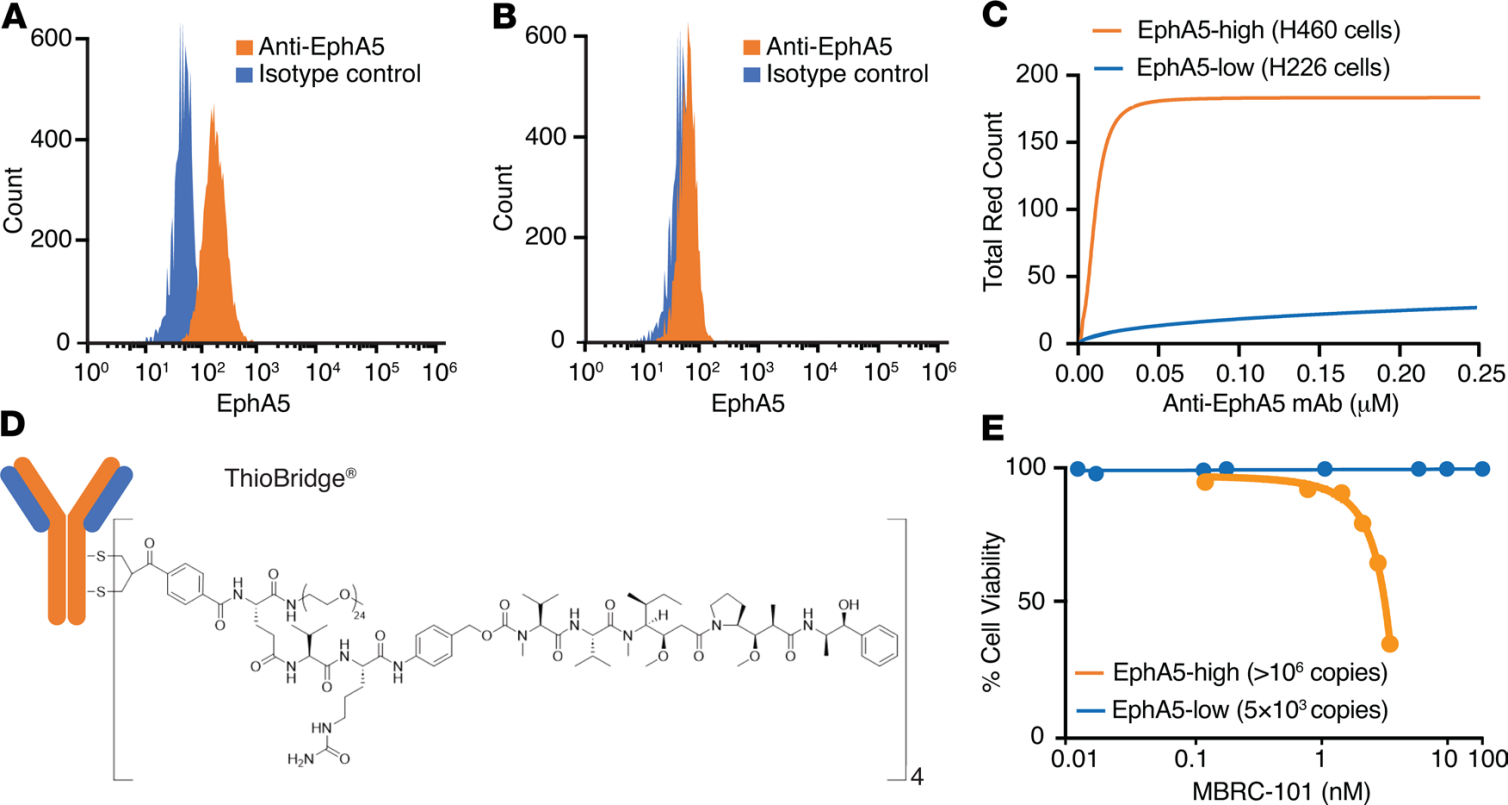
Binding specificity of the anti-EphA5 antibody and cytotoxicity of MBRC-101 in cells. (**A** and **B**) Flow cytometry analysis shows EphA5 expression on the surface of well-established lung cancer cells. (**C**) Receptor-mediated antibody internalization in EphA5-positive cells (H460). Internalization was not detected in EphA5-low cells (H226). (**D**) MBRC-101 consists of a humanized IgG1 mAb linked to MMAE, its cytotoxic payload, through a ThioBridge bis-sulfone conjugation unit. This unit rebridges the interchain disulfides of the antibody and incorporates a protease-cleavable linker, resulting in an ADC with a homogeneous DAR4. (**E**) MBRC-101 kills EphA5^+^ cells in a concentration-dependent manner.

The function and specificity of the anti-EphA5 antibody were next evaluated in a receptor-mediated internalization assay. Expression of endogenous EphA5 on the surface of representative lung cancer cells (H460 and H226 cells) was confirmed by flow cytometry (Figure 3, A and B). Internalization of the complex EphA5/anti-EphA5 antibody in H460 cells resulted in the rapid release of fluorogenic signals caused by exposure to a low pH environment, thereby confirming (a) that receptor-mediated internalization occurred and (b) that it was processed via acidic lysosomes and endosomes (pH 4.5–5.5). Internalization was not detected in control EphA5-low cells (H226) (Figure 3C), again indicating specificity.

### MBRC-101 is a first-in-class ADC targeting EphA5-expressing solid tumors.

Given the favorable features of the EphA5 target and the anti-EphA5 mAb, we generated an ADC (termed MBRC-101) to treat EphA5-expressing human cancers. The conjugation technology resulted in an ADC with homogeneous DAR4 (in contrast to vedotin-based ADC agents, which are heterogeneous conjugates) and improved stability over maleimide-based ADC agents (Figure 3D) (18). The binding affinity of MBRC-101 to EphA5 was characterized by using a surface plasmon resonance binding assay (Biacore), and it was determined to be equivalent in value to the unconjugated antibody (i.e., unconjugated antibody KD = 2.38 × 10^–9^ M; MBRC-101 KD = 2.05 × 10^–9^ M). Cytotoxicity of MBRC-101 in vitro was assessed by cell-killing assays in representative cells expressing high levels of EphA5 (> 1 × 10^6^ copies) and found to be concentration dependent and proportionate to the level of EphA5 on the cell surface (Figure 3E).

### MBRC-101 is highly efficacious at inducing tumor regression in vivo.

**Figure 4:**
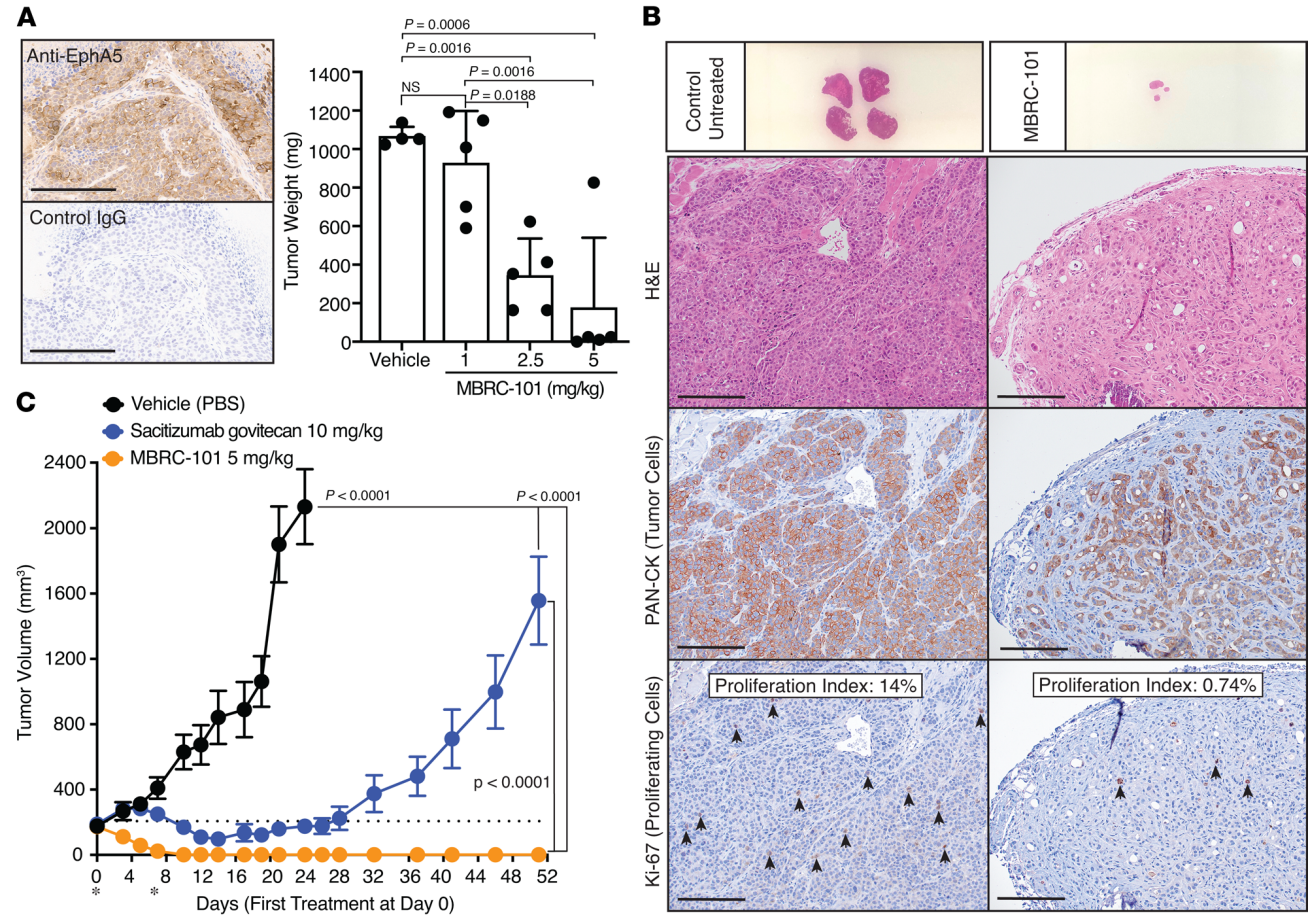
MBRC-101 antitumor activity. (**A**) IHC of tissue sections from a PDX model of TNBC shows moderate and heterogenous expression of EphA5. Scale bar, 200 μm. Weight of tumors collected at the end of the dose-range-finding study confirmed antitumor activity of MBRC-101 at clinically relevant dose levels. Data are presented as means ± SEM. Statistical tests were ordinary 1-way ANOVA coupled with post hoc Tukey’s multiple comparisons test. (**B**) Histological analysis of tissue sections of tumors collected at the end of treatment. The pan-tumor marker PAN-CK was used to detect cancer cells, and Ki-67 served to detect proliferating cancer cells (arrows). The Ki-67 proliferation index was assessed by point counting from 500 to 1,000 cells and reported as percent positive cells. Scale bar, 20 μm. (**C**) MBRC-101 antitumor activity compared with sacituzumab govitecan, an FDA-approved ADC for the treatment of TNBC. Data are presented as means ± SEM. Statistical tests were 2-way ANOVA coupled with post hoc Tukey’s multiple comparisons test among treatment groups.

The efficacy of MBRC-101 was first tested in vivo by using an index PDX tumor model of TNBC. We used IHC to confirm the expression of EphA5 in this model (Figure 4A) and sequencing analysis to confirm the presence of the intact epitope recognized by MBRC-101 (not shown). IHC staining determined that EphA5 expression in this TNBC PDX model was moderate and heterogenous, with most tumor cells displaying cytoplasmic staining, though some tumor cells displayed a clear membranous staining pattern (Figure 4A). Next, we performed a dose-range-finding study when tumors reached approximately 170–200 mm^3^ in average group volume. A total of 2 treatments were given on days 1 and 7 at 1 mg/kg, 2.5 mg/kg, and 5 mg/kg. Tumors were measured 3 times per week. Mice treated with 1 mg/kg MBRC-101 remained in the study for at least 30 days, and mice treated with 2.5 mg/kg and 5 mg/kg MBRC-101 completed the study at 52 days. We observed partial tumor regression at both 1 mg/kg and 2.5 mg/kg; complete tumor regression (without tumor regrowth) was obtained at 5 mg/kg (Figure 4A). Body weights of mice were not affected by treatment with MBRC-101 (Supplemental Figure 2A). Histological analysis with a pan-cytokeratin (PAN-CK) marker detected the presence of tumor cells in remaining tumor sections (Figure 4B). However, the proliferation index, according to the marker Ki-67, indicated that cancer cells from the treated tumors were not proliferative (Figure 4B).

Using the same PDX model of TNBC, we next compared the antitumor activity of MBRC-101 with that of sacituzumab govitecan, an FDA-approved ADC for the treatment of TNBC (19, 20), as a positive comparator used in this context to help the assessment of MBRC-101 efficacy and perhaps better predict its potential effectiveness in patients. Expression of TROP2, the target of sacituzumab govitecan, was confirmed with a standardized IHC methodology (membrane H-score = 230). MBRC-101 outperformed sacituzumab govitecan, showing complete and sustained regression for all treated tumors (Figure 4C), with no effect on body weight (Supplemental Figure 2B).

**Figure 5:**
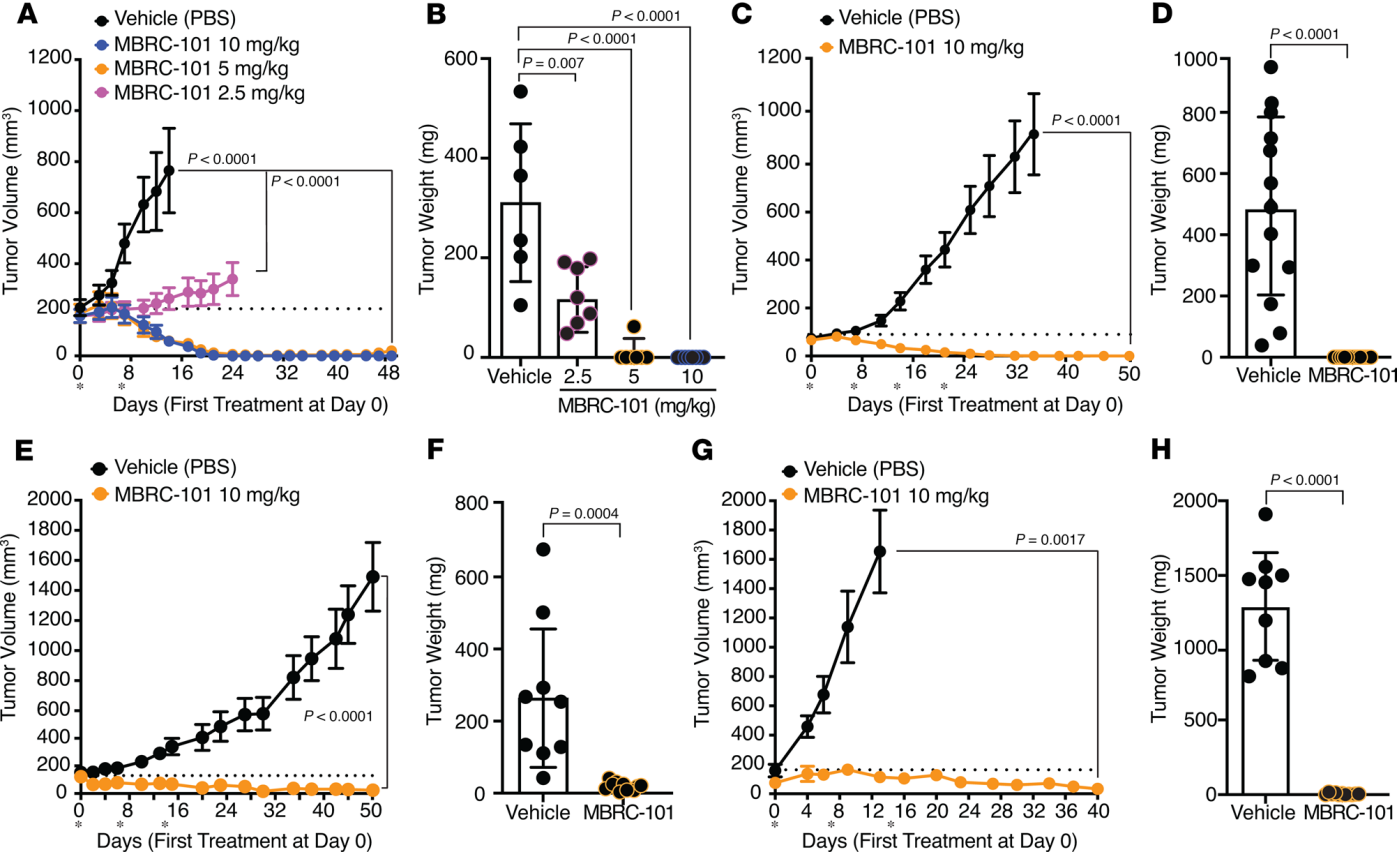
Antitumor activity of MBRC-101 in various PDX models of human cancers. (**A** and **B**) Head-and-neck squamous carcinoma (ordinary 1-way ANOVA and Tukey’s multiple comparisons test). (**C** and **D**) Lung squamous cell carcinoma (unpaired *t* test). (**E** and **F**) Lung adenocarcinoma (unpaired *t* test). (**G** and **H**) TNBC (unpaired *t* test). Treatments were given weekly for 2 weeks and up to 4 weeks (asterisks). In all cases, complete tumor regression without regrowth was achieved at 5 mg/kg or 10 mg/kg, with no observed weight loss. All data are presented as means ± SEM.

We tested MBRC-101 in several other PDX tumor models of aggressive human cancers, such as squamous head and neck cancer (Figure 5, A and B), squamous cell lung cancer (Figure 5, C and D), lung adenocarcinoma (Figure 5, E and F), and a second PDX model of TNBC (Figure 5, G and H). In all cases, MBRC-101 induced complete tumor regression at dose levels of 5 mg/kg or 10 mg/kg. Consistently, body weights of treated mice were not affected by treatment (Supplemental Figure 2, C–F). Finally, MBRC-101 was evaluated in 3 well-established cell-derived (CDX) models of lung cancer (Supplemental Figure 3). In all cases, MBRC-101 either delayed tumor growth or partially or completely regressed tumors without affecting the apparent overall health of the mice.

### Toxicokinetics of MBRC-101 in Sprague-Dawley rats and cynomolgus monkeys.

**Figure 6:**
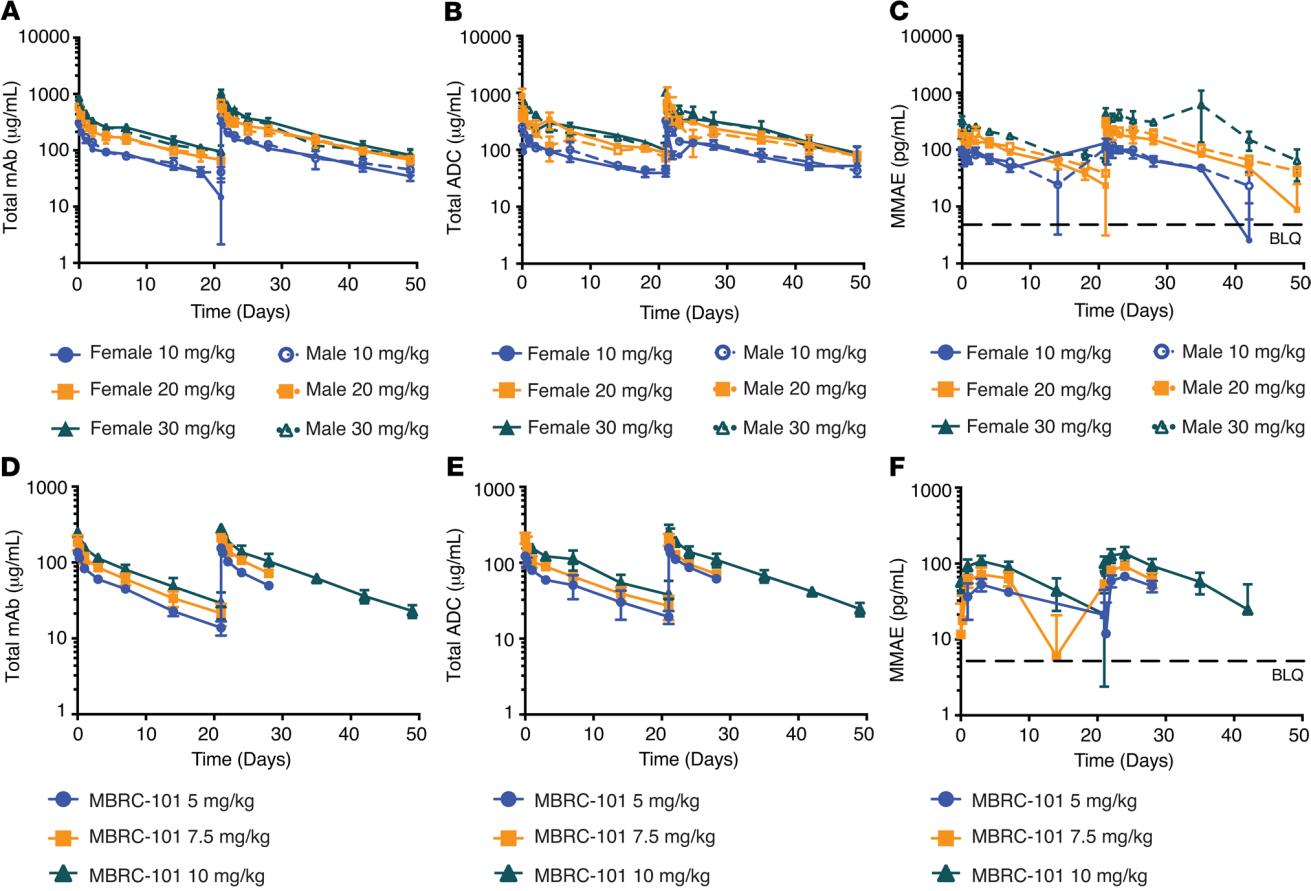
Toxicokinetics of MBRC-101 in rats and monkeys. (**A**) Total mAb, (**B**) total ADC, and (**C**) unconjugated MMAE concentrations in Sprague-Dawley rats after administration of MBRC-101 via i.v. bolus (days 1 and 22) at doses of 10, 20, and 30 mg/kg. (**D**) Total mAb, (**E**) total ADC, and (**F**) unconjugated MMAE concentrations in cynomolgus monkeys after administration of MBRC-101 via i.v. bolus (days 1 and 22) at doses of 5, 7.5, and 10 mg/kg. All data are presented as means ± SD. BLQ, below the limit of quantification.

Serum toxicokinetic profiles of MBRC-101 in rats (Figure 6, A–C) and monkeys (Figure 6, D–F) showed a biphasic decline, consistent between species. Systemic exposure of total ADC, total mAb, and unconjugated MMAE increased with higher dose level in a generally proportional manner (Tables 1 and 2). No apparent accumulation occurred after the second dose, and no remarkable differences occurred between male and female animals except for MBRC-101 on days 1 and 22 in the rat 20 mg/kg dose-level group. Mean half-life values for the total ADC ranged from 193 hours to 471 hours in rats and 175 hours to 269 hours in monkeys. Unconjugated MMAE was detected only for approximately 168 hours after each dose (Tables 1 and 2, and Figure 6, C and F).

### Toxicology findings were considered off target and attributed to the MMAE payload.

Repeated dose i.v. studies (*n* = 2 individual doses, 3 weeks apart with up to a 4-week recovery period) were conducted in Sprague-Dawley rats and cynomolgus monkeys. MBRC-101 was well tolerated in cynomolgus monkeys up to 10 mg/kg, which was the highest nonseverely toxic dose. In both species, no on-target toxicities were identified for any tissue, including those that normally express EphA5. All findings were consistent with previously described off-target toxicities attributed to MMAE (21). Toxicologic findings are summarized in Table 3.

In Sprague-Dawley rats, MBRC-101 was well tolerated at all dose levels (≤ 30 mg/kg) and a severely toxic dose was not reached. Clinical signs were limited to focal swelling of the head and/or cheek area (severity but not incidence increased with dose) and occasional observations of abrasions or scabs that lacked a dose-proportional response. Dose-dependent histologic findings attributed to MBRC-101 occurred in the liver, bone marrow, eye, lung, testis/epididymis, thymus, and mammary gland. Testicular degeneration resulting in fewer luminal sperm and more cellular debris in the epididymis along with sperm granulomas was present at all dose levels. In the lung, an increased number of alveolar macrophages (some degenerate) and alveolar epithelia hyperplasia were present in both sexes at ≥ 10 mg/kg, and minimal mononuclear cell infiltrates were present at 20 mg/kg and 30 mg/kg. Liver findings included hepatocellular necrosis associated with hemorrhage, increased number of mitotic figures in the sinusoidal lining cells, and minimal apoptosis or necrosis of the biliary epithelium at ≥ 20 mg/kg. These findings correlated with dose-dependent mild to moderate increases in serum alanine aminotransferase, aspartate aminotransferase, and total bilirubin levels, and dose-dependent minimal to mild increases in levels of alkaline phosphatase, cholesterol, and triglycerides. There was an increase in the number of mitotic figures and a minimal increase in apoptosis at ≥ 20 mg/kg in the cornea; however, ophthalmologic examinations were unremarkable in all animals. Decreased bone marrow cellularity correlated with dose-dependent peripheral decreases in neutrophils (mild to marked), eosinophils (moderate to marked), and RBC mass (minimal to mild) at ≥ 20 mg/kg. Minimal to mild, dose-dependent decreases in lymphocytes were present at ≥ 10 mg/kg and were attributed to decreased cellularity in both bone marrow and thymus.

In monkeys, no MBRC-101–related effects on clinical signs, body weight, food consumption, ophthalmologic examination, and electrocardiography were identified at doses up to 10 mg/kg. Target organs included testes, ovary, cornea, and bone marrow; however, testicular degeneration and ovarian degeneration were noted only in the dose-range-finding study, in which sexually mature animals were used. Few vacuolated and/or shrunken hypereosinophilic cells were present in the limbus of the cornea at the 10 mg/kg dose. Based on both histologic and cytologic evaluation of the bone marrow, the primary effects attributed to MBRC-101 included dose-dependent decreases in numbers of neutrophils (mild to marked) and eosinophils (moderate to marked) at ≥ 7.5 mg/kg, and decreases in RBC mass (minimal to mild) at all dose levels.

## Discussion

In this study, we introduce EphA5 as a molecular target for antibody-based therapies and present the preclinical development of a first-in-class ADC to treat EphA5-expressing solid tumors. Previous work from our group demonstrated the selective expression of EphA5 in NSCLC and identified it as an important regulator of radiation resistance through its role in DNA damage repair (11). Our current findings suggest that EphA5 is a broad, but specific, molecular target in multiple human malignancies.

We conducted detailed structural biology studies to map the epitope of our EphA5-targeted monoclonal antibody, gaining insights into its binding profile relative to other members of the Eph and ephrin ligand–receptor families. We designed and developed MBRC-101, a first-in-class ADC, which consists of a humanized mAb conjugated to an MMAE payload via a valine-citrulline cleavable linker through the ThioBridge disulfide rebridging conjugation technology, thereby resulting in a homogeneous DAR with improved stability (18) compared with FDA-approved, vedotin-based ADC agents such as brentuximab, enfortumab, and polatuzumab (21, 22).

In vitro and in vivo studies confirmed that MBRC-101 binds specifically to EphA5 and exhibits antitumor activity across multiple human solid tumor models at various levels of antigen expression and clinically relevant doses. In preclinical studies, MBRC-101 demonstrated potent antitumor activity similar to other ADC agents used in solid tumors (5, 21, 22) but with apparent superior toxicokinetics and tolerability. Preclinical models of aggressive human tumors, including several CDXs and PDXs, revealed that the efficacy of MBRC-101 is not limited by tumor heterogeneity or antigen expression levels. Complete tumor eradication and partial regressions with sustained tumor growth delay were observed in all tested models, even at low doses (e.g., 1 mg/kg). These results suggested that MBRC-101 may benefit from the well-documented bystander effect of MMAE-based ADC agents, particularly in the context of heterogeneous tumors with immune-cell infiltration (23–25). Concordantly, and despite our efforts to characterize the expression of EphA5 in solid human tumors and PDX models, a clear correlation between EphA5 expression, whether membranous or cytoplasmic, and tumor response has yet to be established. As we continue to study EphA5 as a promising ADC target and refine our strategy to select patient populations more likely to benefit from MBRC-101, a potential correlation between EphA5 levels of expression, its localization in cells, and the suitability of our detection method will become increasingly important. This remains an area of active investigation as we continue to evaluate clinical samples that will provide real-world insight into our clinical trial program.

Notably, MBRC-101 outperformed sacituzumab govitecan in a PDX model of TNBC, demonstrating superior antitumor efficacy in the setting of a clear and contemporary unmet clinical need. Toxicology studies conducted in rats and monkeys demonstrated a favorable safety profile. All findings were considered off target and consistent with other MMAE-based ADC agents (26–29). A severely toxic dose for MBRC-101 was not reached in rats at doses up to 30 mg/kg, and the highest nonseverely toxic dose in monkeys was 10 mg/kg, higher than reported for most MMAE-based ADC agents (21).

This enhanced tolerability is likely attributable to the ThioBridge rebridging conjugation strategy. Indeed, consistent with the anticipated greater stability of the conjugation used, MMAE exposure as a percentage of total antibody of MBRC-101 in monkeys was roughly only one-third that of other ADC linker chemistries that use native cysteine residues. Dose-normalized MBRC-101 total antibody C_max_ and AUC_0–168h_ in monkeys were within the ranges reported for other MMAE-conjugated ADC agents (21, 30–32). Similar pharmacokinetics among ADC agents, including MBRC-101, suggest that distribution and clearance are DAR, linker, and target independent, at least at doses greater than 1 mg/kg used in general toxicity studies in monkeys. Therefore, the greater payload stability of MBRC-101, combined with consistent pharmacokinetics, may provide an improved therapeutic window over current marketed ADC agents.

With its favorable safety profile, MBRC-101 is under evaluation in a first-in-human, multicenter phase 1/1b clinical trial for patients with advanced, metastatic solid tumors refractory to standard treatments (ClinicalTrials.gov, NCT06014658). In conclusion, EphA5 is a viable therapeutic target for several malignant solid tumors. The favorable preclinical attributes of MBRC-101 underscore the potential for EphA5-directed therapies to emerge as a new class of agents for treatment-refractory solid tumors.

## Methods

### Sex as a biological variable.

Our study examined male and female animals. In general, the toxicology findings were similar for both sexes; however, the magnitude and severity of the findings showed some variability between the sexes. No remarkable differences occurred in toxicokinetic parameters between male and female animals.

### IHC of archival human tissues, cynomolgus monkey tissues, and PDX samples.

We performed IHC stainings on a Leica BOND III automated staining platform. Testing for EphA5 used a commercial rabbit polyclonal primary antibody (Novus Biologicals; catalog NBP1-53105) at 3 μg/mL dilution for 1 hour at room temperature (RT) followed by the BOND Polymer Refine Detection Kit for detection in FFPE tissues. We used the rabbit IgG (DA1E) isotype-matched negative control (Cell Signaling Technologies; catalog 3900) to determine any nonspecific staining inherent in the detection reagents or tissues and to define any potential background reactivity from those sources. All de-identified human tumor tissues were commercially obtained from the Discovery Life Sciences tissue bank. An FDA-recommended normal tissue array (Quickarrays; catalog MNO961) was used to evaluate EphA5 expression in normal human adult tissues. The percentage of EphA5^+^ cells was determined based on EphA5 expression analysis conducted by board-certified pathologists.

### MPA.

We conducted the MPA screening at Integral Molecular (17). In brief, we performed the optimization of antibody-binding conditions in HEK293 cells transfected with human EphA5-encoding plasmids, protein A, or vector alone. After optimization, we performed the antibody binding to the MPA protein library (*n* >6,000 human membrane protein clones). Briefly, HEK293 cells expressing each MPA protein clone were arrayed in duplicates in a matrix format for high-throughput screening. We added the anti-EphA5 antibody to the MPA at the predetermined concentrations and measured binding across the protein library on an Intellicyt iQue by using a fluorescent-labeled secondary antibody (Jackson Immunoresearch; catalog 109-606-008). Each array plate contained both positive (Fc-binding) and negative (empty vector) controls to ensure plate-to-plate reproducibility. Antibody interactions with any off-targets identified by the MPA screening were further confirmed in a second flow cytometry experiment using serial dilutions and sequencing to reverify target identity.

### Structural visualization and analysis of EphA5.

We used TOPCONS (33) to predict the consensus protein topology (i.e., amino acid residues corresponding to extracellular, transmembrane, and intracellular regions) of human EphA5. Three-dimensional, atomic-level structural visualization and analysis were performed with UCSF ChimeraX, version 1.7 (34). Given that the only 2 experimentally determined structures of EphA5 in the Protein Data Bank (PDB) comprise residues N59–S235 (PDB ID 4et7) (16) and residues P653–P939 (PDB ID 2r2p), we retrieved a high-fidelity predicted structure of full-length human EphA5 (corresponding to UniProt accession P54756) from the AlphaFold Protein Structure Database (35, 36). For structural visualization, regions containing residues with low or very low confidence scores (i.e., predicted local distance difference test score < 70) were not shown. For structural comparison with EphA5 orthologs, we retrieved high-fidelity, predicted, full-length structures of rhesus monkey (F7GJT5), cynomolgus monkey (A0A2K5W6J6), and rat (P54757) from the AlphaFold Protein Structure Database. Finally, we superimposed the structured extracellular region (residues S58–P561) or epitope (residues R306–E330) of human EphA5 onto the corresponding region of each ortholog according to best fit, using the Matchmaker function in UCSF ChimeraX; settings included the Needleman-Wunsch sequence alignment algorithm, BLOSUM-62 matrix, and a gap penalty of 1. An root mean square deviation value for all C_α_ atom pairs was calculated for each comparison.

### Flow cytometry.

For cell-binding assays, cells were resuspended in FACS buffer (PBS containing 1% BSA plus 0.1% sodium azide) and seeded in 96-well U-bottom plates at 10^5^ (100 μL) cells per well. Cell pellets were incubated in the presence of either the custom humanized anti-EphA5 primary antibody or an isotype control (ThermoFisher Scientific; catalog 02-7102) at 4°C for 30 minutes. After the incubation, cells were washed twice with FACS buffer and resuspended in Alexa Fluor 647 anti-human antibody (Invitrogen; catalog A-21445) diluted to 5 μg/mL in FACS buffer. After incubation, cells were washed twice with FACS buffer. We measured MFI in the Attune NxT Flow Cytometer with 10,000 gated events acquired per sample. Live cell populations were gated based on the forward and side-scatter dot plot, allowing the Alexa Fluor 647 MFI values to be measured in the red laser channel. The number of EphA5 copies on the cell surface was determined by quantitative FACS (Bangs Laboratories).

### Antibody humanization and conjugation methods.

Design of Composite Human Antibody (Abzena) variable regions of the structural models of the murine monoclonal antibody 11C12 (11) V regions were produced by using the PDB Viewer and analyzed to identify constraining residues in the V regions that were likely to be essential for the binding properties of the antibody. Variant sequences were analyzed for the occurrence of potential T cell epitopes as determined by application of a proprietary in silico technology. The ThioBridge-VCP-MMAE linker-payload was custom synthesized by Abzena and conjugated as described (18) to the humanized anti-EphA5 antibody to prepare highly monomeric (>95%) ADC batches, with DAR4 being the most abundant species present (DAR4 > 95%; DAR0 was not detected).

### Internalization and cell-killing assays.

Cells were commercially obtained from the American Tissue Type Collection. We used the Incucyte real-time live-cell analysis and the Incucyte Fabfluor-pH systems to study antibody-mediated internalization of EphA5 in cancer cells naturally expressing distinct levels of EphA5. The anti-EphA5 antibody was conjugated to a pH-sensitive fluorescent probe (Incucyte Fabfluor-pH Antibody Labeling Dye) according to manufacturer’s instructions. We acquired live cell images every 30 minutes and used the Incucyte’s proprietary analysis software (Incucyte Cell-by-Cell Analysis Software) to analyze the data. Two independent cell-killing technologies were used to assess MBRC-101 cytotoxicity: live-cell analysis of cell confluence and the CellTiter-Glo luminescent cell viability assay with a starting seeding density of 4,000 cells/well and exposure to increasing concentrations of the ADC for 5 days. Killing assays were performed with Chinese hamster ovary cells (which naturally have low levels of surface EphA5) engineered to express high levels (> 1× 10^6^ copies) of human EphA5.

### Efficacy studies in patient-derived and CDX tumor xenograft models.

We tested the antitumor activity of MBRC-101 in vivo in PDX models purchased from The Jackson Laboratory and maintained and propagated in our animal facilities. The TNBC model TM00098 was derived from the primary site (breast) of an grade 3 invasive ductal carcinoma that was treatment naive at the time of surgical sample collection. EphA5 IHC staining had a membrane H-score of 55, with 5% of cells scoring at 1+ intensity; 10% of cells scoring at 2+, and 30% of cells scoring at 3+. The head-and-neck cancer model TM01141 was obtained from the primary site of a head-and-neck squamous cell carcinoma of unknown grade, smoking history, or previous treatments. EphA5 IHC staining had a membrane H-score of 120, with 10% of cells scoring at 1+ intensity, 10% of cells scoring at 2+, and 30% of cells scoring at 3+.

The TNBC PDX model TM00096 was derived from the lung metastasis lesion of a grade 3 invasive ductal carcinoma. The sample was treatment naive at the time of collection. EphA5 IHC staining had a membrane H-score of 18, with 2% of cells scoring at 1+ intensity, 5% of cells scoring at 2+, and 2% of cells scoring at 3+. Two lung cancer PDX models (TM00219 and TM00226) were obtained from female former smokers with lung adenocarcinoma. TM00226 was obtained from the primary site (lung) and had a EphA5 membrane H-score of 45, with 20% of cells scoring at 1+ intensity, 5% of cells scoring at 2+, and 5% of cells scoring at 3+. TM00219 was obtained from a lymph node metastasis and had an EphA5 membrane H-score of 95, with 15% of cells scoring at 1+ intensity, 10% of cells scoring at 2+, and 20% of cells scoring at 3+. The lung cancer model TM00188 was derived from the primary site of a male patient who was a former smoker with lung squamous cell carcinoma, grade 3, and an EphA5 membrane H-score of 29, with 10% of cells scoring at 1+ intensity, 2% of cells scoring at 2+, and 5% of cells scoring at 3+.

Tumor fragments were implanted s.c. into the right flank of female mice (*n* = 5, minimum, per mouse cohort) and allowed to grow to 170–200 mm^3^. Tumor measurements were performed 2–3 times per week with the aid of a digital caliper. Body weights of mice were measured prior to study initiation, at each measurement day, and at the end of the study. The weight of excised tumors was measured at the end of the study. CDX models were obtained after s.c. injection of human cancer cells in the flank of NOD-SCID mice (Charles River). Male and female mice were used.

### Dose-range-finding study in cynomolgus monkeys.

Twelve cynomolgus monkeys, 7–9 years of age and weighing 4.2–10.1 kg, were assigned to 4 dose groups that received 0, 5, 10, or 15 mg/kg MBRC-101 by i.v. bolus. Each group contained 3 monkeys (*n* = 1 or 2 males and females each) that were dosed 3 weeks apart and necropsied 3 weeks after the second dose.

### Toxicology and toxicokinetic studies in Sprague-Dawley rats and cynomolgus monkeys.

Repeated dose toxicology studies in Sprague-Dawley rats and cynomolgus monkeys were conducted according to GLP guidelines to assess the potential toxicity and toxicokinetics of MBRC-101 when administered by i.v. bolus 3 weeks apart, followed by a 4-week recovery period.

Sprague-Dawley rats were assigned to 2 separate animal cohorts for assessment of toxicity and toxicokinetics, with dose groups receiving 0, 10, 20, or 30 mg/kg MBRC-101. Rats in the toxicity cohort were necropsied 1 week after receiving the second dose (*n* = 10 per sex per group) or at the end of a 4-week recovery period (*n* = 5 rats per sex per group at 0 and 30 mg/kg). Rats in the toxicokinetic group were dosed solely for the purpose of blood collection at established time points from pre-dose through the end of the recovery period. Time points collected after each dose administration included 0.25, 6, 24, 48, 96, 168, and 336 hours. Additional samples were collected at 504 hours and 672 hours after the second dose.

Thirty-two cynomolgus monkeys, 31–38 months of age and weighing 1.9–3.3 kg, were assigned to 4 dose groups that received 0, 5, 7.5, or 10 mg/kg MBRC-101 by i.v. bolus. Twenty-four monkeys (*n* = 3 per sex per group) were necropsied 1 week after the second dose, and 8 monkeys were necropsied at the end of a 4-week recovery period (*n* = 2 per sex per group for 0 and 10 mg/kg). Blood samples were collected throughout the study at the same time points as in the rat study. Samples collected prior to each dose were analyzed for the presence of antidrug antibodies.

Parameters evaluated for each study included clinical observation, food consumption, body weight, electrocardiology testing (monkeys only), ophthalmologic examination, clinical pathology, gross necropsy findings, organ weight, and histopathology. Clinical pathology assessment consisted of hematology, clinical chemistry, urinalysis, coagulation, and cytologic evaluation of bone marrow (monkeys only).

### Bioanalytical analysis of GLP-compliant studies.

In rat and monkey sera, quantification of total ADC (DAR ≥ 1) and total mAb (DAR ≥ 0) was determined by ELISA. Anti-MMAE (Acro Biosystems; catalog MME-M5252) and recombinant human EphA5 (R&D Systems; catalog 3036-A5) were used as the capture antigens for the total ADC and total mAb assays, respectively. For both assays, anti–human IgG HRP (BD Pharmigen; catalog 555788) served as the detection antibody. The determination of unconjugated MMAE in both rat and monkey serum was performed by liquid chromatography– tandem mass spectrometry with deuterium-labeled MMAE as the internal standard. All assays were validated according to the International Conference on Harmonization Harmonized Guideline M10. Concentrations less than the lower limit of quantitation for rat (total mAb = 0.05 μg/mL; total ADC = 0.25 μg/mL; and unconjugated MMAE = 30 pg/mL) or monkey (total mAb = 0.25 μg/mL, total ADC = 0.15 μg/mL, and unconjugated MMAE = 30 pg/mL) were set to zero for toxicokinetic analysis. Toxicokinetic parameters were calculated by noncompartmental analysis by using Phoenix WinNonlin 64 software (Version 8.3.5; Certara Companies).

### Statistics.

Data for the antitumor activity of MBRC-101 in multiple PDX and CDX models are reported as mean ± SEM. Ordinary 1-way ANOVA coupled with post hoc Tukey’s multiple comparisons test or Dunnett’s multiple comparisons test, and 2-way ANOVA coupled with post hoc Tukey’s multiple comparisons test and unpaired *t* test were performed by using GraphPad Prism 10 (Figures 4 and 5, and Supplemental Figure 3). *P* values < 0.05 were considered statistically significant.

### Study approval.

Mice (male and female) were obtained commercially from The Jackson Laboratories or Charles River and were housed in specific pathogen- and opportunist-free rooms at a controlled temperature (20 ± 2°C), humidity (50% ± 10%), light/dark cycle (light, 0700–1900 hours; dark, 1900–0700 hours), and access to food and water ad libitum at the research animal facilities of the Rutgers Cancer Institute. The mice were cared for in compliance with all applicable laws and guidelines, including those from the U.S. Department of Health and Human Services, Public Health Service, and the Office of Laboratory Animal Welfare. The IACUC of the Rutgers Cancer Institute and the Rutgers New Jersey Medical School approved all animal experiments, and the Rutgers Animal Facility followed guidelines as set forth by the Association for Assessment and Accreditation of Laboratory Animal Care (AAALAC) International.

Studies in rats (male and female) and monkeys (male and female) were conducted at Inotiv and at the Keeling Center for Comparative Medicine and Research of the University of Texas M.D. Anderson Cancer Center. Both facilities were fully accredited by the AAALAC International and registered with and inspected by the U.S. Department of Agriculture. Procedures were reviewed and approved by the IACUC at each facility and complied with the Animal Welfare Act and the *Guide for the Care & Use of Laboratory Animals: Eighth Edition*.

### Data availability.

All data associated with this study are present in the article, the Supplemental Figures, and the Supporting Data Values file.

## Author contributions

IC, WA, and RP jointly supervised this work and contributed equally to the manuscript. FIS, KFB, IC, WA, and RP designed research studies. FIS, FHFT, VDO, SYK, ERC, CM, and DIS conducted experiments, acquired data, and analyzed data. FIS, CM, KFB, SCA, IC, WA, and RP wrote the manuscript. All authors edited the manuscript.

## Supplementary Material

Supplemental data

Supporting data values

## Figures and Tables

**Table 3:**
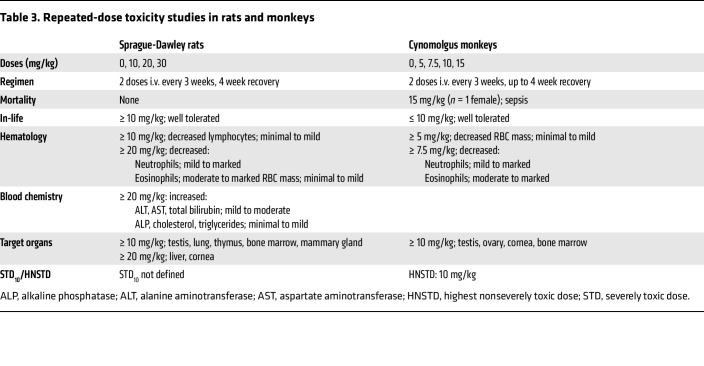
Repeated-dose toxicity studies in rats and monkeys

**Table 2:**
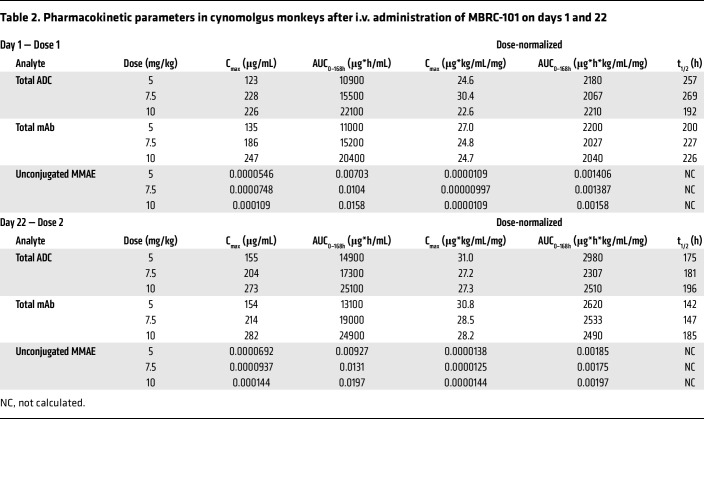
Pharmacokinetic parameters in cynomolgus monkeys after i.v. administration of MBRC-101 on days 1 and 22

**Table 1:**
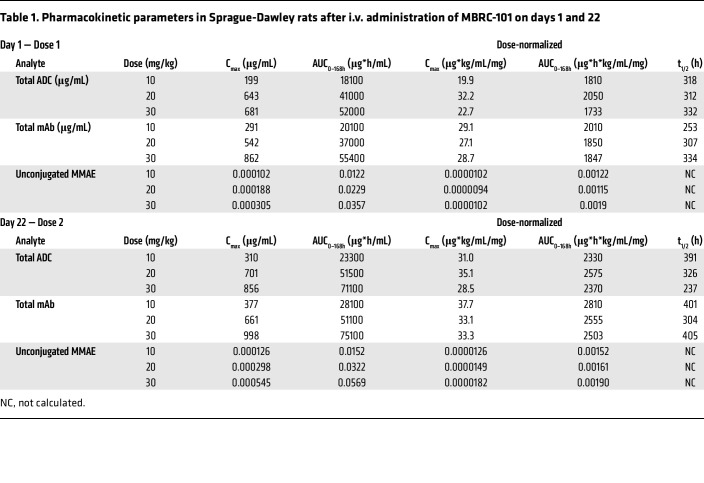
Pharmacokinetic parameters in Sprague-Dawley rats after i.v. administration of MBRC-101 on days 1 and 22

## Version 1 07/15/2025

Electronic publication

## References

[1] Colomer R (2001). Herceptin: from the bench to the clinic. Cancer Invest.

[2] Lambert JM, Chari RV (2014). Ado-trastuzumab Emtansine (T-DM1): an antibody-drug conjugate (ADC) for HER2-positive breast cancer. J Med Chem.

[3] Cardillo TM (2011). Humanized anti-Trop-2 IgG-SN-38 conjugate for effective treatment of diverse epithelial cancers: preclinical studies in human cancer xenograft models and monkeys. Clin Cancer Res.

[4] Starodub AN (2015). First-in-human trial of a novel Anti-Trop-2 antibody-SN-38 conjugate, sacituzumab govitecan, for the treatment of diverse metastatic solid tumors. Clin Cancer Res.

[5] Breij EC (2014). An antibody-drug conjugate that targets tissue factor exhibits potent therapeutic activity against a broad range of solid tumors. Cancer Res.

[6] de Bono JS (2019). Tisotumab vedotin in patients with advanced or metastatic solid tumours (InnovaTV 201): a first-in-human, multicentre, phase 1-2 trial. Lancet Oncol.

[7] Challita-Eid PM (2016). Enfortumab vedotin antibody-drug conjugate targeting nectin-4 is a highly potent therapeutic agent in multiple preclinical cancer models. Cancer Res.

[8] Rosenberg J (2020). EV-101: A phase I study of single-agent enfortumab vedotin in patients with nectin-4-positive solid tumors, including metastatic urothelial carcinoma. J Clin Oncol.

[9] Ab O (2015). IMGN853, a folate receptor-α (FRα)-targeting antibody-drug conjugate, exhibits potent targeted antitumor activity against FRα-expressing tumors. Mol Cancer Ther.

[10] Moore KN (2017). Safety and activity of mirvetuximab soravtansine (IMGN853), a folate receptor alpha-targeting antibody-drug conjugate, in platinum-resistant ovarian, fallopian tube, or primary peritoneal cancer: a phase I expansion study. J Clin Oncol.

[11] Staquicini FI (2015). Receptor tyrosine kinase EphA5 is a functional molecular target in human lung cancer. J Biol Chem.

[12] D’Angelo S (2018). Selection of phage-displayed accessible recombinant targeted antibodies (SPARTA): methodology and applications. JCI Insight.

[13] Arvanitis D, Davy A (2008). Eph/ephrin signaling: networks. Genes Dev.

[14] Chen X (2016). EphA5 protein, a potential marker for distinguishing histological grade and prognosis in ovarian serous carcinoma. J Ovarian Res.

[15] Giaginis C (2010). Clinical significance of ephrin (eph)-A1, -A2, -a4, -a5 and -a7 receptors in pancreatic ductal adenocarcinoma. Pathol Oncol Res.

[16] Huan X (2013). Unique structure and dynamics of the EphA5 ligand binding domain mediate its binding specificity as revealed by X-ray crystallography, NMR and MD simulations. PLoS One.

[17] Tucker DF (2018). Isolation of state-dependent monoclonal antibodies against the 12-transmembrane domain glucose transporter 4 using virus-like particles. Proc Natl Acad Sci U S A.

[18] Badescu G (2014). Bridging disulfides for stable and defined antibody drug conjugates. Bioconjug Chem.

[19] Bardia A (2019). Sacituzumab Govitecan-hziy in Refractory Metastatic Triple-Negative Breast Cancer. N Engl J Med.

[20] Wahby S (2021). FDA approval summary: accelerated approval of sacituzumab govitecan-hziy for third-line treatment of metastatic triple-negative breast cancer. Clin Cancer Res.

[21] Neff-LaFord HD (2024). The vedotin antibody-drug conjugate payload drives platform-based nonclinical safety and pharmacokinetic profiles. Mol Cancer Ther.

[22] Chen H (2017). Tubulin inhibitor-based antibody-drug conjugates for cancer therapy. Molecules.

[23] Okeley NM (2010). Intracellular activation of SGN-35, a potent anti-CD30 antibody-drug conjugate. Clin Cancer Res.

[24] Li F (2016). Intracellular released payload influences potency and bystander-killing effects of antibody-drug conjugates in preclinical models. Cancer Res.

[25] Zhou J (2019). Immunogenic cell death in cancer therapy: Present and emerging inducers. J Cell Mol Med.

[26] Heiser RA (2024). Brentuximab vedotin-driven microtubule disruption results in endoplasmic reticulum stress leading to immunogenic cell death and antitumor immunity. Mol Cancer Ther.

[27] Dumontet C (2023). Antibody-drug conjugates come of age in oncology. Nat Rev Drug Discov.

[28] Tarantino P (2023). Optimizing the safety of antibody-drug conjugates for patients with solid tumours. Nat Rev Clin Oncol.

[29] Donaghy H (2016). Effects of antibody, drug and linker on the preclinical and clinical toxicities of antibody-drug conjugates. MAbs.

[30] https://astellas.us/docs/PADCEV_label.pdf.

[31] https://www.gene.com/download/pdf/polivy_prescribing.pdf.

[32] https://labeling.pfizer.com/ShowLabeling.aspx?id=20632.

[33] Tsirigos KD (2015). The TOPCONS web server for consensus prediction of membrane protein topology and signal peptides. Nucleic Acids Res.

[34] Goddard TD (2018). UCSF ChimeraX: meeting modern challenges in visualization and analysis. Protein Sci.

[35] Jumper J (2021). Highly accurate protein structure prediction with AlphaFold. Nature.

[36] Varadi M (2022). AlphaFold Protein Structure Database: massively expanding the structural coverage of protein-sequence space with high-accuracy models. Nucleic Acids Res.

