# Supplementary material for: First-generation and preclinical evaluation of an EphA5-targeted antibody-drug conjugate in solid tumors
Source: J Clin Invest. 2025 Jul 15;135(14):e188492. doi: 10.1172/JCI188492 (PMC12259246; doi:10.1172/JCI188492)
Supplement: Supplemental data [file jci-135-188492-s242.pdf]

A

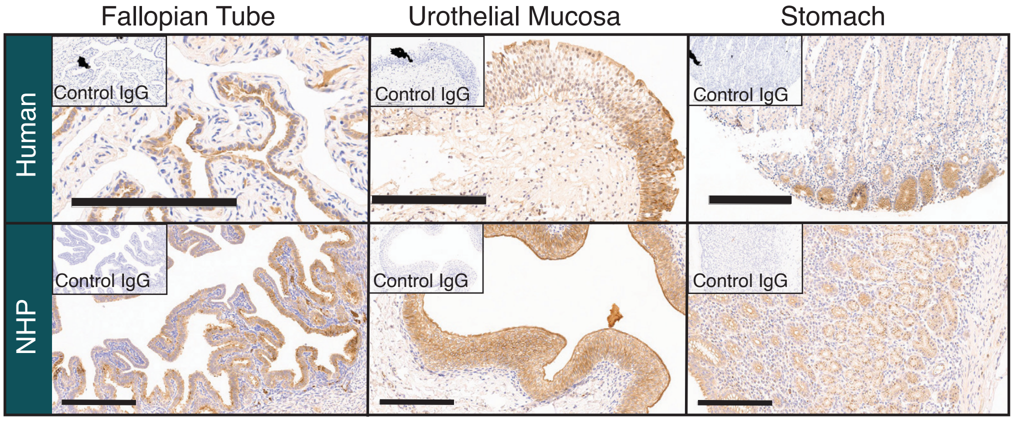

B

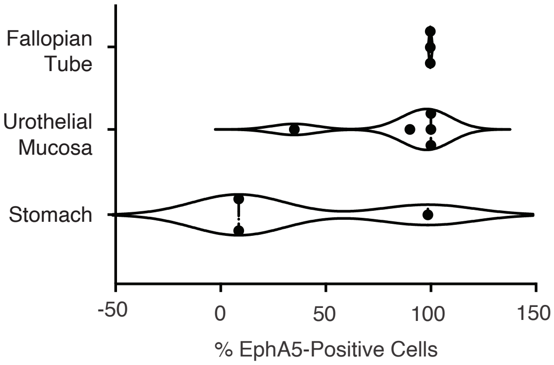

Supplemental Figure 1. (**A**) **Illustrative images of EphA5 expression in normal human and NHP tissues**. Both cytoplasmic and membrane staining were detected. Human urothelial mucosa and stomach: scale bar, 300 μm. Human fallopian tube and NHP tissues: scale bar, 200 μm. (**B**) Percentage of EphA5-expressing cells in normal tissues.

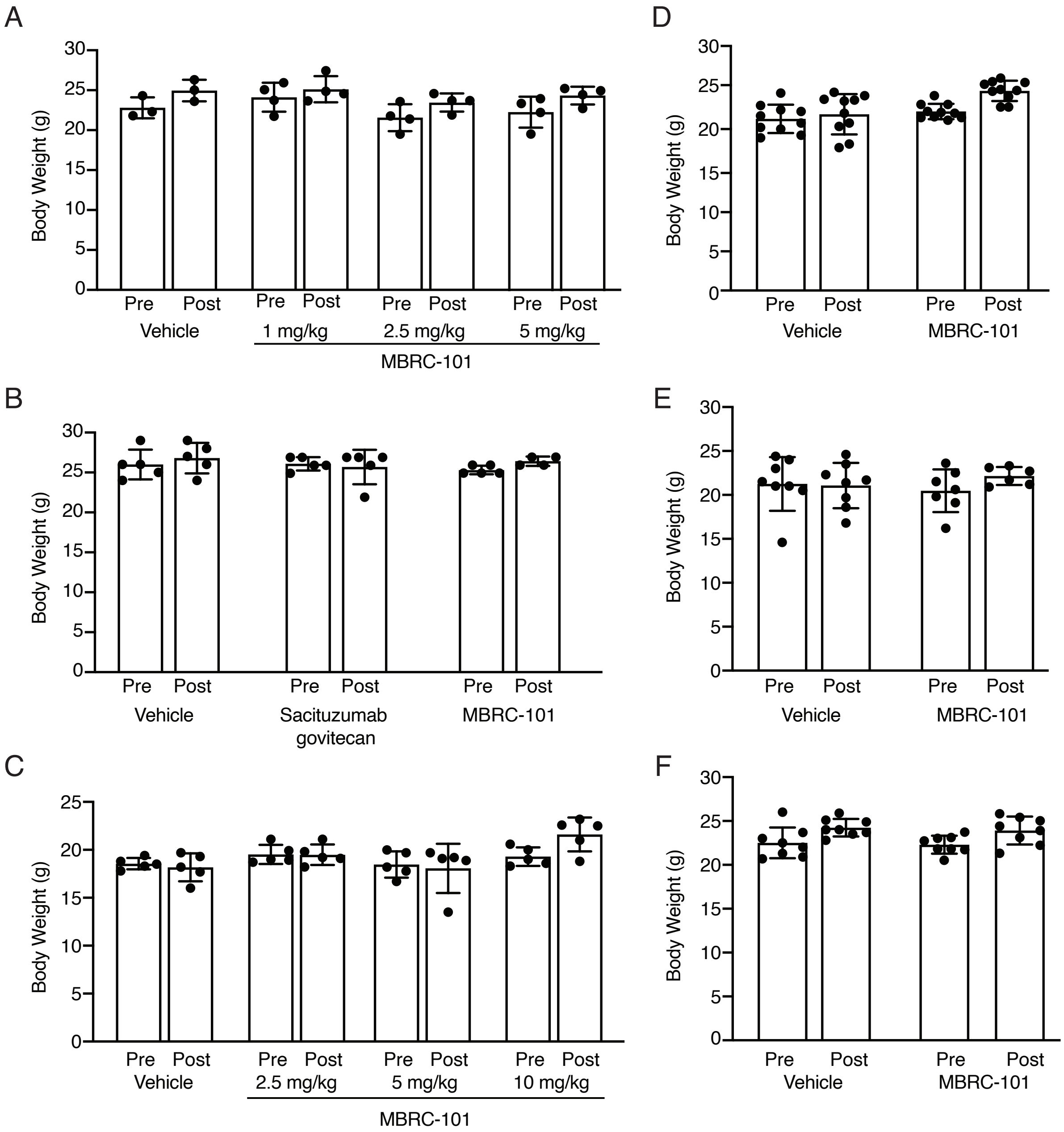

Supplemental Figure 2. Body weight of mice pre- and post-treatment with MBRC-101 in various efficacy studies. Treatment with saline was used as negative control. All data presented as means ± SEM.

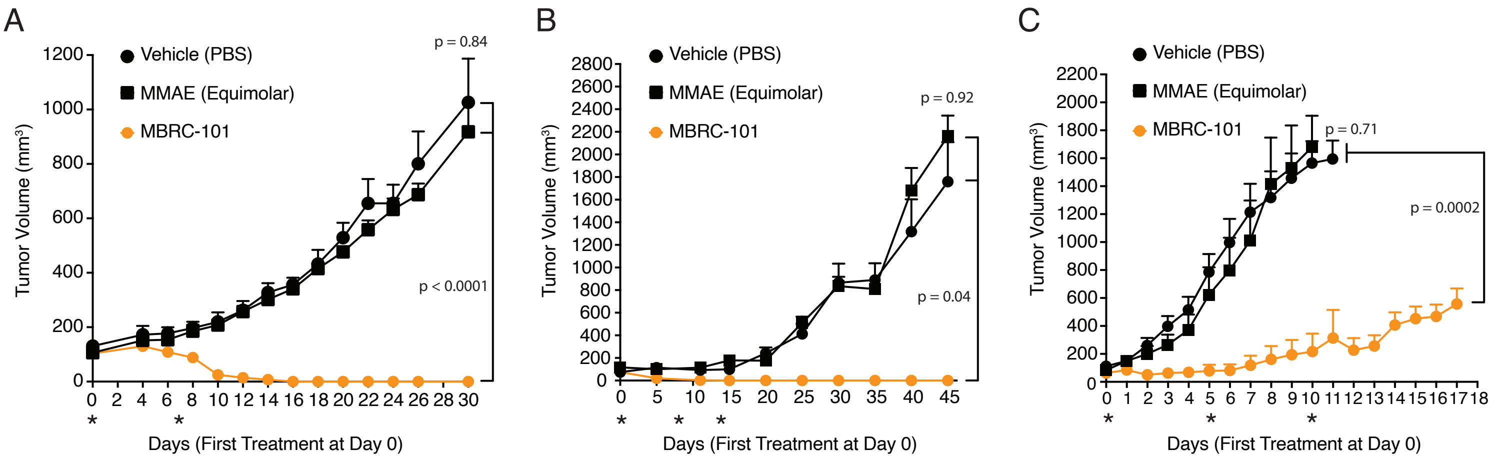

Supplemental Figure 3. **Anti-tumor activity of MBRC-101 in CDX models of lung cancer**. (**A**) A549 human lung adenocarcinoma cell-derived tumors. Ordinary one-way ANOVA coupled with Dunnett's multiple comparisons test. (**B**) H522 human lung adenocarcinoma cell-derived tumors. Ordinary one-way ANOVA coupled with Dunnett's multiple comparisons test. (**C**) H460 human large cell lung cancer cell-derived tumors. Ordinary one-way ANOVA coupled with Dunnett's multiple comparisons test. Treatments were given weekly for 2 or 3 weeks (*). All data presented as means ± SEM.
